# Large asymmetry in the magnetoresistance loops of ferromagnetic nanostrips induced by Surface Acoustic Waves

**DOI:** 10.1038/s41598-021-88113-x

**Published:** 2021-04-21

**Authors:** David Castilla, Manuel Muñoz, Miguel Sinusía, Rocío Yanes, José L. Prieto

**Affiliations:** 1 Instituto de Sistemas Optoelectrónicos y Microtecnología (ISOM), Universidad Politécnica de Madrid, Avda. Complutense 30, 28040 Madrid, Spain; 2 Instituto de Tecnologías Físicas y de la Información (CSIC), Serrano 144, 28006 Madrid, Spain; 3 Dpto. Física Aplicada, University of Salamanca, Plaza de los Caídos S/N, 37008 Salamanca, Spain

**Keywords:** Materials science, Ferromagnetism, Spintronics

## Abstract

In this work we show that Surface Acoustic Waves (SAW) can induce a very large asymmetry in the magnetoresistance loop of an adjacent ferromagnetic nanostrip, making it look as if it had exchange bias. The Surface Acoustic Wave induces a DC voltage in the ferromagnetic nanostrip. For measurements at constant current, this DC voltage makes the AMR loop asymmetric. In a series of different electrical experiments, we disentangle two different contributions to the induced DC voltage. One of them is independent on the external magnetic field and it is likely due to the acoustoelectric effect. A second contribution depends on the external magnetic field and it is a rectified voltage induced in the piezoelectric substrate as a response to the magnetization dynamics in the magnetostrictive nanostrip. The large asymmetry in the magnetoresistance loop reported in this work is a manifestation of an effective transfer of energy from the SAW to the magnetization dynamics, a mechanism that has been very recently appointed as a possible mean to harvest energy from a heat source.

## Introduction

The field of spintronics is in a constant search for efficient ways of manipulate the magnetization in a ferromagnetic material without the need of an external magnetic field. Spin Transfer Torque (STT) allows to manipulate local magnetization with an electric current^1^, but it often requires of a large current density to produce measurable effects, leading to detrimental thermal effects^2,3^. An alternative strategy to manipulate local magnetization is the use of Surface Acoustic Waves (SAW). Almost a decade ago, Weiler et al.^4^ showed that an elastic wave could lead to an acoustic driven ferromagnetic resonance (FMR) in a Nickel film, even at relatively low frequencies. The same authors, using a Co/Pt film, produced a pure spin current with the acoustic FMR and measured magnetoelastic spin pumping^5^. Indeed, acoustic FMR can lead to a sizable magnetization precession angle^4^, comparable to what has been reported in standard spin pumping ferromagnetic resonance experiments^6,7^. Therefore, acoustic energy transferred from the SAW to a magnetic device has been found to produce many interesting results within the field of magnetism and spintronics. To name few, a SAW can assist the switching in ferromagnetic films^8^ or nano-elements^9,10^, reduce the energy required for magnetic recording^11^ or even decrease the energy dissipation in spin-transfer-torque random access memories^12^. SAWs can also assist the domain wall motion^13,14^ and there is a proposal to use SAWs to control the movement of magnetic domain walls (DWs)^15^. In this work, we show that a SAW can produce a large asymmetry in the anisotropic magnetoresistance (AMR) loop of a ferromagnetic nanostrip, making it look like as if it was exchanged biased. We have disentangled the different contributions to the final signal, acoustically induced voltage, Inverse Spin Hall Effect (ISHE) and rectification voltages. We inferred the existence of an additional source of rectified DC voltage, coming from the electric response of the piezoelectric substrate to the magnetoelastic strain generated in the nanostrip by the local radiofrequency (RF) field. The DC voltage generated in the nanostrip due to the SAW is quite large (tens of microvolts) and it is also strongly dependent on the RF power delivered by the SAW. Therefore, the ferromagnetic magnetostrictive nanostrip becomes a non-coherent RF demodulator, with the advantages of ferromagnetic and piezoelectric materials, large thermal stability and radiation resistance. The RF demodulation is also dependent on the external magnetic field, which may add functionality in some practical applications.

## Results and discussion

**Figure 1:**
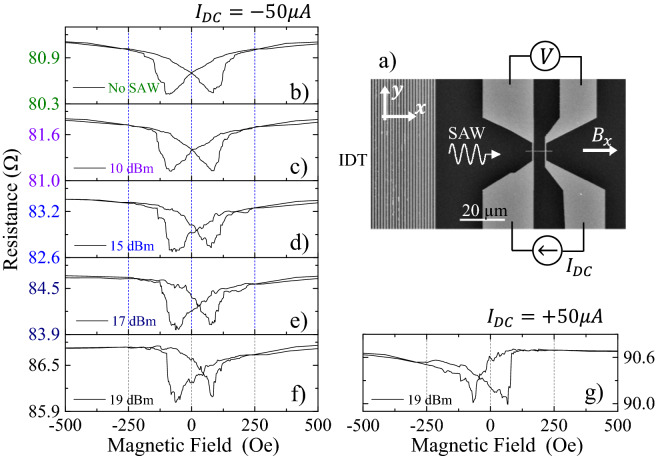
(**a**) SEM photograph of the experimental device, with a IDT to excite the Surface Acoustic Waves (vertical lines visible on the left hand side of the picture) and a ferromagnetic nanostrip aligned with the direction of the travelling SAW. (**b**) to (**f**) AMR loops for different SAW powers showing an increasing asymmetry as the power of the SAW increases. (**g**) Same experiment as in (**f**) but reversing the direction of the measuring DC current.

The experimental set-up used is in this experiment is schematized in Fig. 1a. An Inter-Digitated Transducer (IDT) was deposited over a ScAlN piezoelectric layer. The IDT is designed so the Rayleigh mode of the SAW is found at 1.2 GHz. More technical details of the piezoelectric substrate can be found in Ref.^14^. In the direction of the travelling wave, 40 µm away from the end of the IDT, we place a Ni ferromagnetic nanostrip 10 µm long and 400 nm wide, with the structure Cr(4 nm)/Ni(35 nm)/Pt(2 nm). The ferromagnetic Ni nanostrip was deposited under a magnetic field that induces an anisotropy along the *y*-axis which, together with the shape anisotropy, results in an effective uniaxial anisotropy axis at 55° with the nanostrip axis. The Ni nanostrip is connected to four contact pads, with the structure Cr(20 nm)/Au(120 nm), as visible in Fig. 1a. Further details of the magnetic properties of the ferromagnetic Ni nanostrip and its magnetization process can be found in Supplementary Information S1.

Firstly, we measure the Anisotropic Magnetoresistance (AMR) loop for different SAW powers. A negative DC current (electrons flowing from the left to the right end of the nanostrip as shown in Fig. 1a) of ${I}_{DC}=-50 \, \upmu \text{A}$ is injected in the nanostrip with a Keithley current source and the voltage is monitored while the external field, produced by an electromagnet, is cycled from − 1200 Oe to + 1200 Oe (although only the central part between $\pm 500 \; {\text{Oe}}$ is visible in the loops). The resistance loop for no SAW is a typical butterfly curve shown in Fig. 1b. If we repeat the experiment in the presence of a SAW, the loop becomes increasingly asymmetric as we rise power of the SAW. For the maximum power we can deliver with our RF signal generator, 19 dBm, the AMR loop is unmistakably asymmetric with the crossover displaced towards positive fields and a more gradual approach to saturation for positive fields. It is also important to note that the resistance value at saturation increases as the power of the SAW increases, from about 80.9 Ω for no SAW to 86.5 Ω for a 19 dBm SAW. As a reference, we note that 19 dBm corresponds to 85 MPa of stress delivered to the nanostrip (see conversion in Supplementary Information S1).

If we reverse the direction of the measuring DC current to ${I}_{DC}=+50 \, \upmu \text{A}$, the asymmetry in the AMR loop reverses as well, showing the same features as before, but this time for negative external magnetic fields, as shown in Fig. 1g. It is also noticeable that the value of the resistance a saturation increases further to 90.6 Ω, simply by reversing the direction of the measuring DC current in the presence of the SAW.

**Figure 2:**
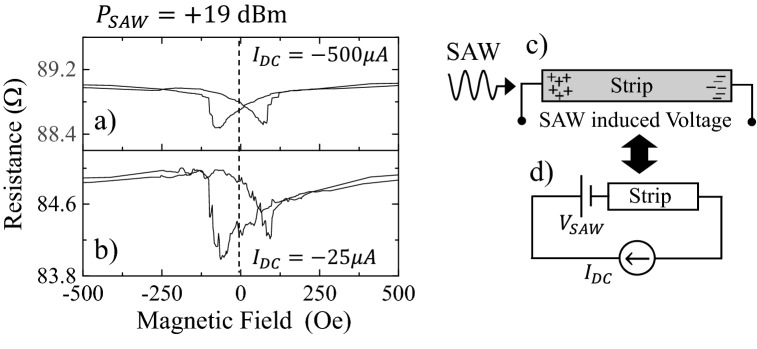
(**a**) AMR loop measured using a DC current of − 500 µA and in the presence of a 19 dBm SAW. (**b**) AMR loop using a DC current of − 25 µA and in the presence of a 19 dBm SAW. (**c**) Schematic representation of the acoustically induced voltage. (**d**) Equivalent circuit when measuring the resistance with a current source in the presence of an acoustically induced voltage.

Figure 2a,b show the AMR loops using ${I}_{DC}=-500 \, \upmu \text{A}$ DC current for the measurement (Fig. 2a) and ${I}_{DC}=-25 \, \upmu \text{A}$ (Fig. 2b), in the presence of a 19 dBm SAW. It becomes clear that, for a constant power delivered to the SAW, the asymmetry of the AMR loop increases as the DC current used in the measurement decreases. Also, the resistance at saturation increases as the current used for the measurement increases. Therefore, the SAW is promoting two distinct effects in the ferromagnetic nanostripe: an increase of the total resistance (noticeable at any external magnetic field) and an asymmetry in the loop. Let us first analyse the origin of the increase in the measured resistance under the action of the SAW.

As the resistance measured at large external magnetic fields (saturation), increases just by changing the direction of the DC current (Fig. 1f,g), we are going to assume that the SAW is inducing a DC voltage with the polarity shown in Fig. 2c,d. In the next paragraph we prove the existence of this voltage and discuss its origin. Let’s first estimate how large would this voltage have to be to account for the changes of resistance we observe in Fig. 1. When using a current source for the measurement of the resistance, the total voltage required to deliver the requested current will not only depend on the Ohmic resistance of the nanostripe, but also on this DC voltage induced by the SAW (V_SAW_). Following the electric circuit depicted in Fig. 2d, the total voltage measured is the addition of the Ohmic voltage drop in the nanostrip plus V_SAW_, which has the same polarity regardless of the direction of the measuring current. For instance, from the measurements in Fig. 1f,g, we can obtain directly the voltage induced by a 19 dBm SAW. For ${I}_{DC}=-50 \, \upmu \text{A}$, we have that $86.7\, \Omega = {R}_{Ni}-{V}_{SAW}/50 \, \upmu \text{A}$. Equally, when using $+50 \, \upmu \text{A}$, if ${V}_{SAW}$ remains with the same polarity, the measured resistance at saturation is $90.7\, \Omega = {R}_{Ni}+{V}_{SAW}/50 \, \upmu \text{A}$. Eliminating ${R}_{Ni}$ from both equations, we arrive to ${V}_{SAW}\approx 100 \, \upmu \text{V}$. As the resistance measured for negative ${I}_{DC}$ is given by ${R}_{Ni}-{V}_{SAW}/{I}_{DC}$, it becomes clear that one should obtain larger values of the resistance for larger negative ${I}_{DC}$, as it is the case in the experiment (see Fig. 2a,b). Notice that ${R}_{Ni}$ is the resistance of the Ni nanostrip when a 19 dBm SAW is present, which is a larger that the resistance with no SAW^16^. The enhanced phonon-electron interaction when the SAW is present, increases the temperature and the Ohmic resistance in the metal (see Supplementary information S2 for further details).

**Figure 3:**
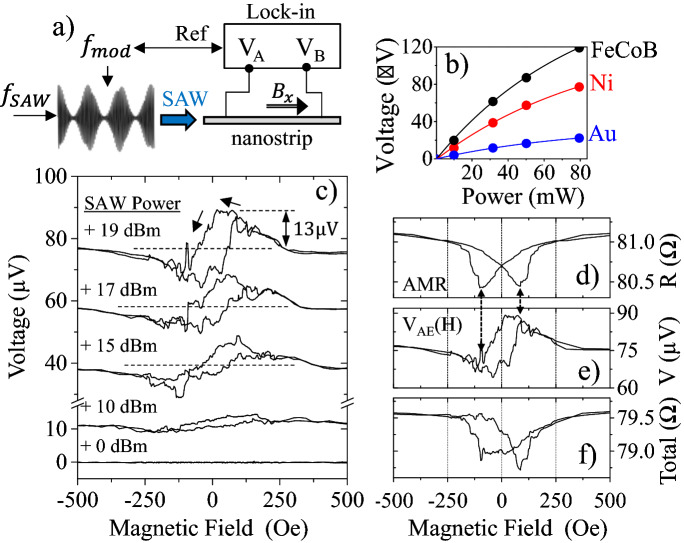
(**a**) Experimental set-up used to measure the acoustic voltage induced by the SAW, using no electric current. (**b**) Voltage induced in three different nanostrips of similar dimensions but made out of different materials, using the experimental set-up described in (**a**). The resistance of the nanostrips is 15 Ω, 80 Ω and 112 Ω for Gold, Nickel and FeCoB, respectively. A 2 nm layer of Pt caps the two ferromagnetic nanostrips. (**c**) Magnetoelastic voltage measured using the set-up described in (**a**) versus external magnetic field for different powers of the SAW. (**d**) AMR loop for no SAW, the same curve as in Fig. 1b. (**e**) Magnetoelastic voltage for 19 dBm, the same curve as the top one in (**c**). (**f**) Sum of the curve in (**d**) and (**e**), turning first (**e**) into resistance by dividing the data by the current used in the AMR measurement, $-50 \, \upmu \text{A}$.

In order to prove the existence of this voltage, we use the alternative experimental set-up displayed in Fig. 3a. The SAW is exited at 1.2 GHz as before, but modulated in amplitude by a lower frequency (400 Hz) sinusoidal signal, at depth of 80%. The voltage induced in the Ni nanostrip is recorded with a Lock-in amplifier locked to the modulating signal of 400 Hz. Now there is no current flowing through the stripe and the measurement is only sensitive to the modulation of any voltage induced by the SAW, ${V}_{SAW}$. In Fig. 3b we show the amplitude measured for different SAW powers and for three different metallic nanostrips of similar dimensions, two of them ferromagnetic. The red points in this figure refer to the Ni nanostrip used in the measurements described above. As it can be seen, for 19 dBm, the voltage measured is $80 \, \upmu \text{V}$, which is precisely 80% of the estimated ${V}_{SAW}$ in the previous paragraph, due to the 80% amplitude modulation of the SAW power. This SAW induced voltage seems to be dependent on the resistance of the nanostrip. The nominal resistances of the three nanostrips used in Fig. 3b are ~ 112 Ω for FeCoB, ~ 80 Ω for Ni and ~ 15 Ω for Au and the voltage induced by the SAW is proportional to these resistances.

We now discuss the origin of this DC voltage induced by the SAW. As the SAW induces a small increase of temperature (see Supplementary Information S2), one possibility is that this DC voltage is due to the Seebeck effect. Nevertheless, the Seebeck coefficients of Nickel and Gold have opposite sign, but the voltage induced by the SAW has the same direction always, irrespective of the metal used. The other possibility is the Acoustoelectric Effect. When a material containing mobile charges is subject to the action of a SAW, the free charges experience a unidirectional net displacement due to the action of the travelling elastic wave (Fig. 2c), leading to a DC electric field^17,18^. The acoustoelectric effect has been observed mainly in semiconductors, although it has been recently observed in a FeCoB ferromagnetic stripe^19^. The theoretical work of Parmenter^18^ on the acoustoelectric effect, predicted an enhancement of the acoutoelectric signal in metals when working in the range of GHz. This may justify the fact that we have such a large signal. Also, the fact that the acoustically induced voltage measured in Fig. 3b is proportional to the resistance of the strip, fits with the acoustoelectric effect as, for a given linear momentum transferred from the SAW to the carriers, a larger voltage can be sustained in a more resistive element.

Going back to the Ni nanostrip, we now explore the origin of the asymmetry in the AMR loops in the presence of a SAW (see for instance Fig. 1f,g). Using the same set-up depicted in Fig. 3a, we now cycle the external magnetic field while performing the experiment. As it can be seen in Fig. 3c, there is a hysteretic signal that gets larger as the power of the SAW increases. The value at saturation (for both positive and negative magnetic field) is the same (the acoustically induced voltage already calculated) and, for smaller magnetic fields between $\pm 250\,  {\text{Oe}}$, there is a hysteretic signal, with the same coercivity of the AMR curves in Fig. 1. In Fig. 3d–f, we can see the AMR curve without any SAW (Fig. 3d, which is the same as Fig. 1b), the voltage generated by the SAW at 19 dBm (Fig. 3e, which is also the top plot in Fig. 3c) and the sum of both of them, once the voltage signal of Fig. 3e was converted into resistance, dividing by the measuring DC current ${I}_{DC}=-50 \, \upmu \text{A}$. As we can see in Fig. 3f, the result closely resembles the measurement in Fig. 1f and it captures the main features of the asymmetry. The actual resistance in Fig. 3f is smaller than the one in Fig. 1f, but only because we have not considered the value of the Ohmic resistance of the nanostrip for 19 dBm SAW, which is about 6 Ω larger than the resistance when there is no SAW, as mentioned previously and explained in Supplementary Material S2.

Therefore, the SAW acting on the Ni nanostrip induces a DC voltage independent of the external field (likely an acoustoelectric voltage) and a DC voltage which is dependent on the external magnetic field as shown in Fig. 3c. We still need to stablish the origin of the magnetic field dependent DC voltage, which could be due to the Inverse Spin Hall Effect (ISHE) or to a rectification effect^20,21^.

Weiler et al.^5^ measured the ISHE induced in a Co/Pt film due to the action of a SAW. The stress carried by the SAW, induces a precession of the magnetization^4^ and the associated pure spin current is converted into a DC voltage at the Pt interface via the ISHE. They named this process Magnetoelastic Spin Pumping. Incidentally, the curve for 19 dBm in Fig. 3c, closely resembles the one measured by Weiler et al. (Fig. 3b in Ref.^5^), although we measure a peak amplitude of 13 µV for 19 dBm, while they measured a much smaller 1 µV peak amplitude using + 30 dBm pulses. Indeed, such a large DC voltage (13 µV) is unlikely the sole consequence of a ISHE voltage, because our stripe is only 400 nm wide and it has only 2 nm of Pt. In order to estimate how much voltage may be induced by Magnetoelastic Spin Pumping via ISHE, we can use the well-known expression that relates the precession cone with DC voltage^22^, ${V}_{ISHE}$

1 $${V}_{ISHE}=RwP(-e{\alpha }_{SH}{\lambda }_{SD}\mathrm{tanh}({t}_{N}/2{\lambda }_{SD})){g}_{\uparrow ,\downarrow }{f}_{SAW}\cdot {\text{sin}}^{2}\Theta $$

We use the resistance of the nanostrip $R=80\,\Omega$, its width $w=400\, \mathrm{nm}$, the frequency of the SAW ${f}_{SAW}=1.2 \, \mathrm{GHz}$, the thickness of the Pt layer ${t}_{N}=2 \, \mathrm{nm}$ and the ellipticity^23^ estimated as $P=0.7$. Also, from a single study for the Ni/Pt interface^7^, we find the spin diffusion length ${\lambda }_{SD}=1.3 \, \mathrm{nm}$, the spin Hall angle ${\alpha }_{SH}=0.11$ and the spin mixing conductance ${g}_{\uparrow ,\downarrow }=4\cdot {10}^{18} {\text{m}}^{-2}$. Finally, we can calculate the precession cone ${\text{sin}}^{2}\Theta$ with the help of micromagnetic simulations (see Ref.^14^ and Supplementary Information S3 for further details). The maximum precession cone calculated for this sample is ${\text{sin}}^{2}\Theta =0.04$. This may seem like a large precession cone in the light of previous works. Incidentally, very recently, an experimental study has visualized in a synchrotron a 25º (peak to peak) oscillation of the magnetization in Ni under the action of a SAW^24^, a similar value to the one we obtain.

With all these data, we calculate a voltage of ${V}_{ISHE}=0.06 \, \upmu \text{V}$, far from the $13.1 \, \upmu \text{V}$ measured. By choosing different values, we could get, at most, a DC voltage due to ISHE in the range of 1 μV. In fact a value of ${V}_{ISHE}=13.1 \, \upmu \text{V}$ for the peak amplitude of the signal at 19 dBm (Fig. 3c), would imply ${\text{sin}}^{2}\Theta >1$ for any credible values of the parameters used in formula^1^. This indicates that the field dependent DC voltage measured in Fig. 3c does not have its origin in the ISHE and it is mostly due to another effect.

A rectification DC voltage in a ferromagnetic resonance measurement, is caused when an RF current flows through a sample where the resistivity depends on the direction of the oscillating magnetization. The resistivity, subject to fluctuations due to AMR, can be described as $\rho \left(m\right)\propto cos\left(\omega t\right)$ and, if an RF current flows through the sample not in phase with the oscillating resistivity ${j}_{RF}cos\left(\omega t+\varphi \right)$, a rectified DC voltage can be induced^21^ as,

2 $${V}_{rect}\propto {j}_{RF}cos\left(\omega t\right)\cdot cos\left(\omega t+\varphi \right)\propto \frac{{j}_{RF}}{2}cos\left(\varphi \right)$$

In any case, $13.1\; \upmu\text{V}$ of rectification signal is also an unusually large value in comparison to the values commonly reported in the bibliography, normally in the range on few μV at most. The total AMR in our Ni samples is about 2% which, with the large precession cone induced by the magnetoelastic field, could imply large variations in the strip resistance of up to ~ 0.4 Ω. Still, the magnetization precession in the nanostrip needs to induce a fairly sizable RF current, to account for the $\sim 13\; \upmu \text{V}$ of rectified DC voltage. For the experiment displayed in Fig. 3a, we do not use a resonant cavity (where electric fields may be present) and we do not use any external current for the measurement. Therefore, a plausible mechanism to induce a RF current in the sample is through induction^21^ (Faraday’s Law). Although, in an idealized picture, the induced eddy current in the Pt and in the Ni layers would have opposite directions, this induced RF eddy current can lead to a nonzero rectification signal because the resistivity of the layers is different.

**Figure 4:**
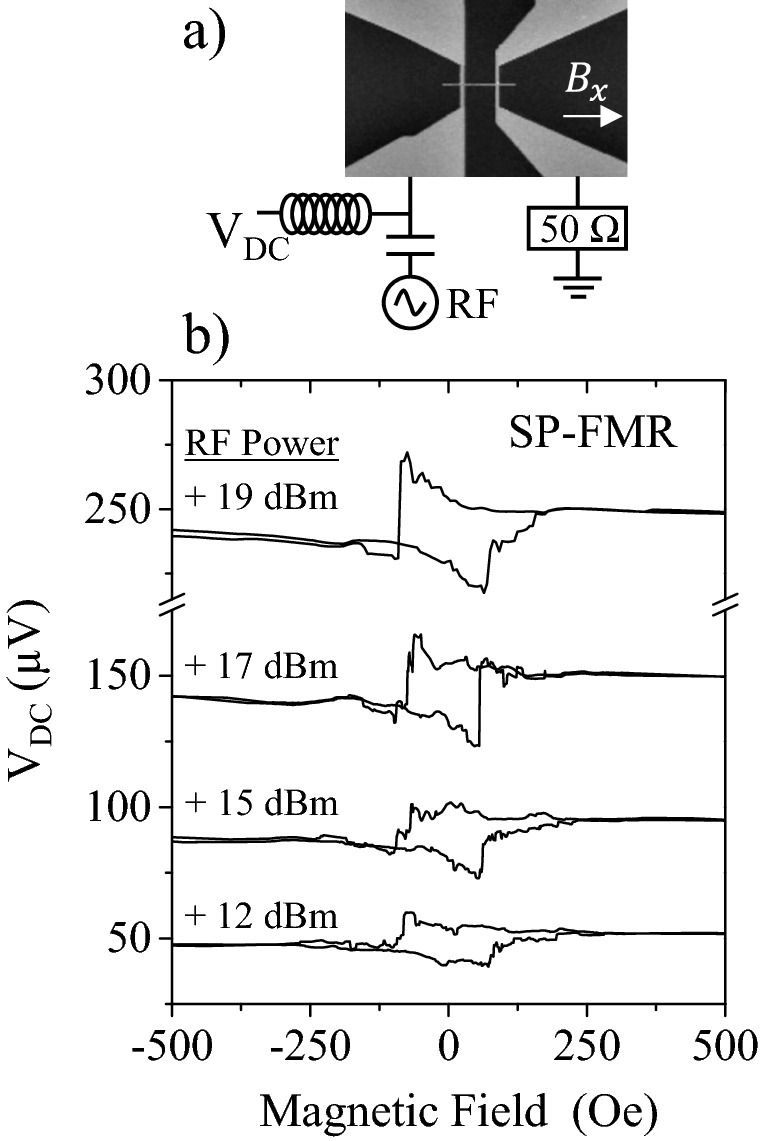
(**a**) Spin Pumping Ferromagnetic Resonance Experiment set up. The RF current is delivered through a Bias Tee and the DC voltage recorded through the DC contact of the Bias Tee. (**b**) Recorded curves for different SAW powers.

In order to have an estimation of how much induced RF eddy current would be required to generate a rectified DC signal in the range of tens of μV, we used a third experimental configuration. Figure 4a shows the set up to measure spin pumping ferromagnetic resonance (SP-FMR). In this experiment, we deliver an RF current to the nanostrip, and measure the induced DC voltage which, for such a narrow strip (400 nm) and for a thin Pt layer, should be mostly a rectification voltage. The results in this experiment are displayed in Fig. 4b. The shape of the curves is not identical to the shape of the curves in Fig. 3c, but it is somehow expected as the RF field in Fig. 3 is magnetoelastic and it points along the *x*-axis, while the RF field in Fig. 4 is an Oersted field and it points along the *y*-axis. In order to comment the main features in Fig. 4 we measured the RF current delivered to the sample by connecting a 50 Ω high frequency oscilloscope in series between the nanostrip and ground. The RF current transmitted for 19 dBm is ${I}_{RF}^{rms}=1.7 \, \mathrm{mA}$. Using Ampère, a rough estimation of the RF Oersted field generated by this current in the 400 × 40 nm^2^ cross section would lead to ${H}_{RF}^{y}\approx 2000 \, \mathrm{A}/\mathrm{m}$. We can now compare this value with the magnetoelastic RF field used in Fig. 3 for 19 dBm

$${H}_{RF}^{x}=\frac{3}{2}\frac{{\lambda }_{S}\cdot {\sigma }_{x}}{{\mu }_{0}{M}_{s}}$$

where we use an isotropic polycrystalline magnetostriction ${\lambda }_{S}=-3.28\cdot {10}^{-5}$, calculated as ${\lambda }_{S}=(2{\lambda }_{100}+3{\lambda }_{111})/5$, ${\mu }_{0}{M}_{S}^{Ni}=0.6 \, \mathrm{ T}$ and ${\sigma }_{x}=85 \, \mathrm{MPa}$ (see Suppl. Info. S1), leading to ${H}_{RF}^{x}\approx 7000 \, \mathrm{A}/\mathrm{m}$. The Oersted RF field in the SP-FMR experiment of Fig. 4 is likely to be smaller than the magnetoelastic RF field induced by the SAW in Fig. 3b, but the signal at coercivity is even larger in the SP-FMR experiment. The most remarkable fact is that, in the SP-FMR experiment there is also a DC voltage at saturation, that increases to almost 250 μV for + 19 dBm, but in the experiment of Fig. 4 there is no SAW applied and we cannot have acoustically induced voltage. We repeated the same SP-FMR experiment, but using a Si/SiO_2_ non piezoelectric substrate, and that DC voltage at saturation is not present (see Suppl. Info. S4). This evidence suggests that the piezoelectric substrate is contributing to the DC rectified voltage. Very recently it has been shown theoretically how the magnetization dynamics of a nanowire on top of a dielectric film, injects SAW underneath^25^. In the experiment in Fig. 4, the vibration induced in the Ni nanostrip by the Oersted RF field, is generating an electric response in the piezoelectric substrate that, if it is not in phase with the precession of the magnetization, it can constitute an additional source of rectified DC voltage.

Disentangling experimentally the precise contribution of the piezoelectric substrate to the DC rectified voltage at any external magnetic field, is not an obvious task. On the other hand, we can establish an additional evidence by changing the sign of the saturation magnetostriction in the nanostrip. In the Ni nanostripe, with negative magnetostriction, a tensile stress produced by the SAW translates into a *perpendicular* magnetoelastic anisotropy in the nanostrip, while if we use a material with positive magnetostriction, a tensile stress produced by the SAW would translate into a *longitudinal* magnetoelastic anisotropy in the nanostrip. Therefore, a change in sign in the magnetostriction should imply a 180° phase shift in the precession of the magnetization, which will be translated on to the rectified signal [see expression^2^].

With this idea, we fabricated another device like the one shown in Fig. 1a but using a 35 nm thick Fe_40_Co_40_B_20_ stripe, deposited on the same ScAlN substrate and with the same buffer (Cr-4 nm) a capping (Pt-2 nm) layers. This time the stripe is 2.5 µm wide in order to improve the signal to noise ratio as this material has a very small AMR (0.1%). Fe_40_Co_40_B_20_ has positive magnetostriction (${\lambda }_{S}=+1.4\cdot {10}^{-5}$) and, with such a small AMR, it is a good material to reduce the usual rectification signals produced in resonant cavities^21^. Also, having a relatively thin Pt layer would decrease the ISHE signal which facilitates the detection of DC voltage generated by other effects. It is also important to note that the FeCoB nanostrip has a uniaxial anisotropy along the nanostrip axis.

**Figure 5:**
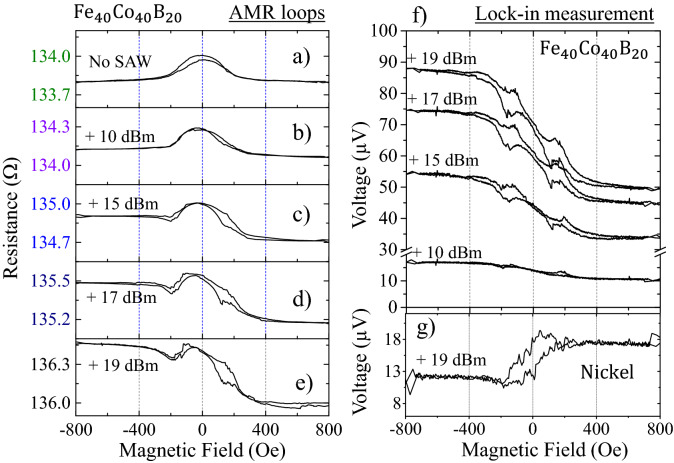
(**a**–**e**) AMR loops in the FeCoB nanostrip with the field applied in the *y*-axis (see Fig. 1a), for increasing power of the SAW as indicated. The loops are measured with de DC current of − 100 µA following the geometry displayed in Fig. 1a. Note the increase in the total resistance when the power of the SAW increases and the large asymmetry generated for large power of the SAW. (**f**) Shows the lock-in measurement in the FeCoB nanostrip following the set-up described in Fig. 3a but with the external field applied along the *y*-axis. (**g**) Shows the lock-in measurement in the Ni nanostripe with the external field along the y-axis (see complete set of measurements in Suppl. Info. S5).

In Fig. 5a–e, we show the AMR loops for the FeCoB nanostrip for selected SAW powers, equivalent to Fig. 1 but with the external magnetic field applied along the *y*-axis. As the FeCoB nanostrip has the anisotropy axis along the nanostrip axis (*x* direction) we need to perform the AMR measurement along the *y*-axis to allow absorption of the magnetoelastic RF field. When the power of the SAW is increased, the overall resistance of the nanostrip increases (acoustically induced voltage) and also, there is an increasing asymmetry in the loop, going from a bell shaped loop for no SAW (Fig. 5a) to a sigmoidal shape for 19 dBm in Fig. 5e. The non-resistive voltage induced by the SAW can be extracted using the set-up displayed in Fig. 3a. This is shown in Fig. 5f for the FeCoB nanostripe. As it can be seen, the voltage induced by the SAW in the FeCoB has the typical sigmoidal shape of a rectified voltage for this configuration (external field perpendicular to the *x*-axis where the DC voltage is measured). This sigmoidal signal, added to the AMR curve without SAW, produces the asymmetric loop displayed in Fig. 5e. This is the same effect we have explained in detail for the Ni nanostrip.

The same measurement was performed for the Ni nanostripe (negative magnetostriction), with the field applied along the *y*-axis, as shown in Fig. 5g for 19 dBm. The complete set of measurements for Ni with the field applied along the *y*-axis, including also SP-FMR, can be found in Suppl. Info. S5. The curves for Ni have opposite symmetry to the ones in FeCoB. While the voltage maximizes for positive fields in the Ni nanostrip (Fig. 5g), it minimizes for positive fields in the FeCoB nanostrip. This indicates that the rectified voltage also depends on the sign of the magnetostriction.

For the SP-FMR experiment (Fig. 4 and Suppl. Info. Fig. S7b,d,f), the precession of the magnetization, phase shifted with the RF current, induces a periodic strain that is transferred to the piezoelectric substrate, giving a rectified DC voltage. In the experiments where the precession is induced by the magnetoelastic interaction with the SAW (Figs. 3c and 5f), the mechanism to generate a large DC voltage is less intuitive but our results are difficult to explain unless there is a source of rectified voltage in the piezoelectric substrate. The SAW induces a precession of the magnetization, not in phase with the stress wave that travels with the SAW, which creates a strain in the ferromagnetic nanostrip, also phase shifted with the SAW, promoting a rectified voltage generated at the piezoelectric substrate. It is a complex mechanism, although plausible in the light of our experiments.

## Conclusions

We have shown that a magnetostrictive ferromagnetic nanostripe under the action of Surface Acoustic Waves, can produce an asymmetric magnetoresistance loop in a standard measuring set-up. We have disentangled the different contributions to the DC voltage generated by the SAW in the nanostrip. There is an induced DC voltage which is independent of the external magnetic field and it is likely caused by the acoustoelectric effect. There is also and induced DC voltage which is dependent on the external magnetic field. This field dependent DC voltage has its origin in the piezoelectric substrate, as a response to the magnetoelastic RF strain induced by the magnetization precession in the ferromagnetic nanostrip.

The large asymmetry in the AMR loops of the nanostrip reported in this work, indicate a very efficient transmission of energy from the SAW to the magnetization dynamics in ferromagnetic nanostrip, inducing a large precession cone. In an article published in parallel with the submission of this manuscript, Kobecki et al*.*^26^ have proposed that the transfer of energy from phonons to magnetization precession can be used as energy harvesting by transferring thermal energy to other excitations. Our work shows that the transfer of energy can be sufficiently relevant to induce very visible effects in the AMR loop. Additionally, the DC signal induced by the SAW is an effective non coherent RF demodulator which converts RF amplitude into a DC voltage that is sensitive to the external magnetic field.

## Supplementary Information

Supplementary Information.
