# Supplementary material for: Large asymmetry in the magnetoresistance loops of ferromagnetic nanostrips induced by Surface Acoustic Waves
Source: Sci Rep. 2021 Apr 21;11:8586. doi: 10.1038/s41598-021-88113-x (PMC8060385; doi:10.1038/s41598-021-88113-x)
Supplement: Supplementary file 1 — Supplementary Information. [file 41598_2021_88113_MOESM1_ESM.docx]

**Large asymmetry in the magnetoresistance loops of ferromagnetic nanostrips induced by Surface Acoustic Waves**

David Castilla^1^, Manuel Muñoz^2^, Miguel Sinusía^1^, Rocío Yanes^3^, José L. Prieto^1*^.

^1^ Instituto de Sistemas Optoelectrónicos y Microtecnología (ISOM), Universidad Politécnica de Madrid, Avda. Complutense 30, 28040 Madrid, Spain.

^2^ Instituto de Tecnologías Físicas y de la Información (CSIC). Serrano 144. 28006, Madrid, Spain.

^3^ Dpto. Física Aplicada. University of Salamanca, Plaza de los Caídos S/N, E-37008, Salamanca, Spain.

**S1. Magnetic details of the Nickel nanostrip**

The Nickel nanostrip is deposited by sputtering and it grows polycrystalline with very small grains in the range of 10-20 nm. The Ni nanostrip has a main uniaxial anisotropy at 55º from the nanostrip axis, which is a consequence of the combination of the polycrystalline texture and the shape anisotropy. A full characterization of the magnetization process of these Nickel nanostrips was done in Ref. 14 of the main text. The nanostrip, at remanence, has a multidomain configuration such as the one shown in Fig. S1 for several SAW powers (see also MFM images in Ref.14 of the main text). The majority of the domains are aligned along the *y*-axis and they are separated by wide pseudo-domains with rotating magnetization along the nanostrip axis. When the external field is applied along the *x*-axis these pseudo-domain walls grow until the cover the entire strip at saturation. Again, more details of the magnetization process can be found in Ref. 14 of the main text, although they are not of particular relevance for the purpose of this work.


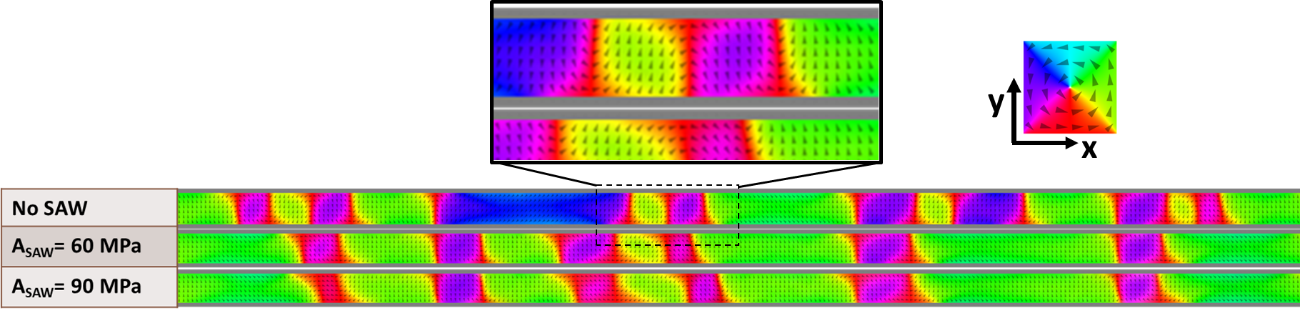


**Figure S1**. Multidomain structure in the Ni nanowire at remanence (H=0) for different SAW powers. Snapshots of the magnetic configuration numerically obtained using Mumax3 code [1].

In order to have an idea of how much stress is delivered from the RF generator to the nanostrip, we measured first the parameter S_11_ of the IDT in a network analyser for its resonance frequency f=1.19197 GHz. We recorded |S_11_(f)|=0.02 (-33 dB), which means that most of the power delivered by the signal generator reaches the IDT. The electromechanical coupling factor is assumed to be 5% from Ref. S2 for a ScAlN device of similar dimensions.

Therefore, for a 1D travelling wave,

$$\frac{1}{2}k^{2}P=\frac{1}{2}A\rho v\xi_{0}^{2}\omega^{2}$$

With *A* the area, *ρ* the density, *v* the velocity of sound, $\xi_{0}$ the amplitude of the wave, $\omega$ the angular frequency, *P* is the electric power and $k^{2}$ the electromechanical coupling coefficient ($k^{2}=0.05)$. The 1/2 on the left side of the formula accounts for the bidirectionality of the IDT. As we know the wavelength of the SAW (spacing of the IDT) and its frequency, we take *v*=λ·f=2.8·10^-6^·1.2·10^9^= 3360 m/s, which is very close to other values reported in the bibliography^3^. The density of ScAlN should be in the range of *ρ*=3500 kg/m^3^ (see Table 1 in Ref.4 of this Supplementary Information). Finally, the area is defined by the width of the IDT (100 µm) and the thickness of the ScAlN (2 µm), so *A*=2·10^-10^ m^2^. With these values, for a +19 dBm power (79.4 mW), we obtain an amplitude of the wave of $\xi_{0}$=0.17 nm. Finally, the amplitude of the stress wave is simply,

$$\sigma_{0}=Y\cdot\frac{2\pi\xi_{0}}{\lambda}$$

Assuming the Young modulus to be *Y*=220 GPa^5^ (our samples have 43% Sc deposited at 500 W from a single target), we calculate a stress of $\sigma_{0}=85.1 \mathrm{MPa}$ for 19 dBm delivered by the RF generator. A very similar value was obtained independently by adjusting the micromagnetic simulations to the experimental results in Ref. 14 of the main text.

We note that the $\sigma_{0}$ we calculate here corresponds mainly to $\sigma_{xx}$. The components $\sigma_{xz}$ and $\sigma_{zz}$ of the Rayleigh wave, have almost negligible influence in the precession, given the dimensions of the strip. In section S4 of this Supplementary Information, we provide more details on this when discussing the amplitude of the precession cone.

There are of course uncertainties in the above calculation. We cannot be certain of the exact value of the electromechanical coupling coefficient, although it should not be far from the value we chose. Also, we have assumed perfect transmission of the stress from the substrate to the Nickel, which is likely not true, given the very different elastic properties of ScAlN and Nickel. Also, we do not know how the ScAlN/Cr/Ni boundary is going to behave in the range of GHz. For instance, if we assume an acoustic reflection coefficient of 0.5 for this boundary, we would need to choose a slightly larger electromechanical coupling coefficient to obtain the 90 MPa that the micromagnetic simulation requires to reproduce the experimental results for 19 dBm.

**S2. Ohmic resistance of the nanostrip when the SAW is acting.**

An alternative method to measure the resistance of the nanostrip, is by delivering an AC current and to measure the voltage generated with a lock-in amplifier phase-locked to the current. As the SAW travels only in one direction and the generated acoustic voltage is DC, this signal gets filtered in the lock-in amplifier. This measurement is therefore insensitive to the SAW induced acoustic voltage and the AMR loop should not show any asymmetry when the SAW is present.

This is clearly visible in Fig. S2. The AMR loop for 19 dBm of SAW shows no asymmetries. Nevertheless, the total resistance does change and, when the SAW is present, the Ohmic resistance is 6 Ω larger than when there is no SAW. As the resistivity varies with temperature as $\rho\left( T \right)=\rho_{0}\left[ 1+\alpha(T-T_{0}) \right]$ and the temperature coefficient for Nickel is *α*=0.006 K^-1^, we can infer an increase of temperature of about 10 K with the maximum power delivered to the SAW. This is due to an enhanced phonon-electron interaction promoted by the SAW, which is visible experimentally as an increase of the resistance or the temperature. When the power of the SAW increases to large values, part of the acoustic loss produces incoherent phonons (thermoelastic or Akhieser effect).

In Fig. 3b of the main text, for large powers of the SAW, we lose the linearity of the curve. According to Parmenter’s work (Ref. 18 in the main text), the acoustically induced voltage should be linear with the power of the acoustic wave. The lack of linearity may be partly due to some Seebeck voltage but, for the three materials we use Au contacts and we should expect different Seebeck behaviour for each of them. As the trend for the three materials is very similar, we believe the loss of linearity may be due to part of the energy of the SAW being lost producing incoherent phonons. These would contribute to an increase of the temperature (and resistance) of the nanostrip and not to acoustically induced voltage.


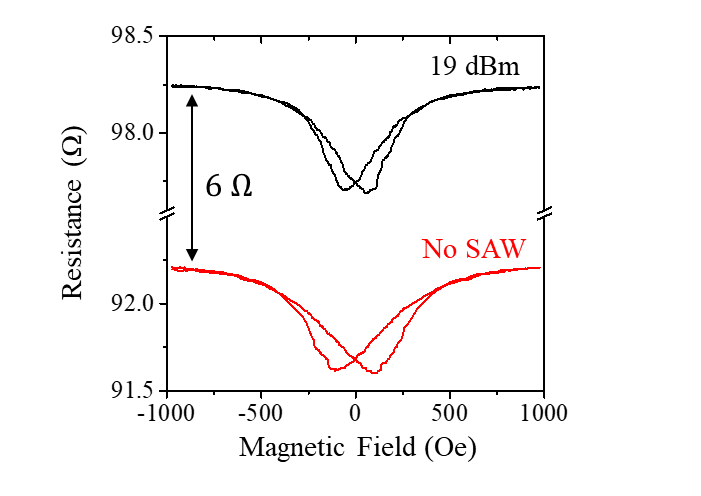


**Figure S2**. AMR loops measured with an AC technique without a SAW (red) and with a 19 dBm SAW. Notice the 6 Ω difference in the Ohmic resistance.

**S3. Calculation of the precession cone**

In order to analyse the effect of SAW on the magnetization precession of our Ni sample, we have performed micromagnetic simulations using Mumax3 code. The dimension of the simulated system are similar to the experimental device (10 μm × 400 nm × 35 nm). The strip was discretized in 2048 × 64 × 1 cells with a cell size of 4.95 × 6.25 × 35 nm^3^. The polycrystalline grain structure was modelled via a Voronoi tessellation of the system in polygonal regions of average diameter d = 20 nm. A nominal value Ms = 2.38x10^5^ A·m^−1^ with a 4% dispersion among grains is chosen for the saturation magnetization. The exchange constant is A=1.05x10^-11^J·m^−1^ and a 20% reduction at the grain boundaries is assumed. Also, we assumed a small anisotropy K_y_=6,0x10^3^ J·m^−3^, similar to those used in Ref. 14 in the main text.

Numerically, the precession cone was obtained in the following way. First, for each value of the magnetic field the magnetization configuration was obtained by cycling the hysteresis loop to that value of the field. In the case of remanence and coercive field, the magnetic configuration is divided in multiple magnetic domains. Therefore, we need to evaluate the initial orientation of the magnetic moments in the sample and take that configuration as reference, $\vec{m}_{0}\left( \vec{r} \right)$. Secondly, for each discretization cell we evaluate the magnetization at a time t, $\vec{m}_{t}\left( \vec{r} \right)$. Obtaining the angle evolved by the magnetization in each cell as: $cos\theta\left( \vec{r},t \right)=\vec{m}_{0}\left( \vec{r} \right)\cdot\vec{m}_{t}\left( \vec{r} \right)$. Finally, we can estimate numerically the precession angle as the spatial average of angle $\theta\left( \vec{r},t \right)$. See also the movie in Supplementary Information.

**Figure S3**. Visualization of how the average angle of the precession cone is calculated.
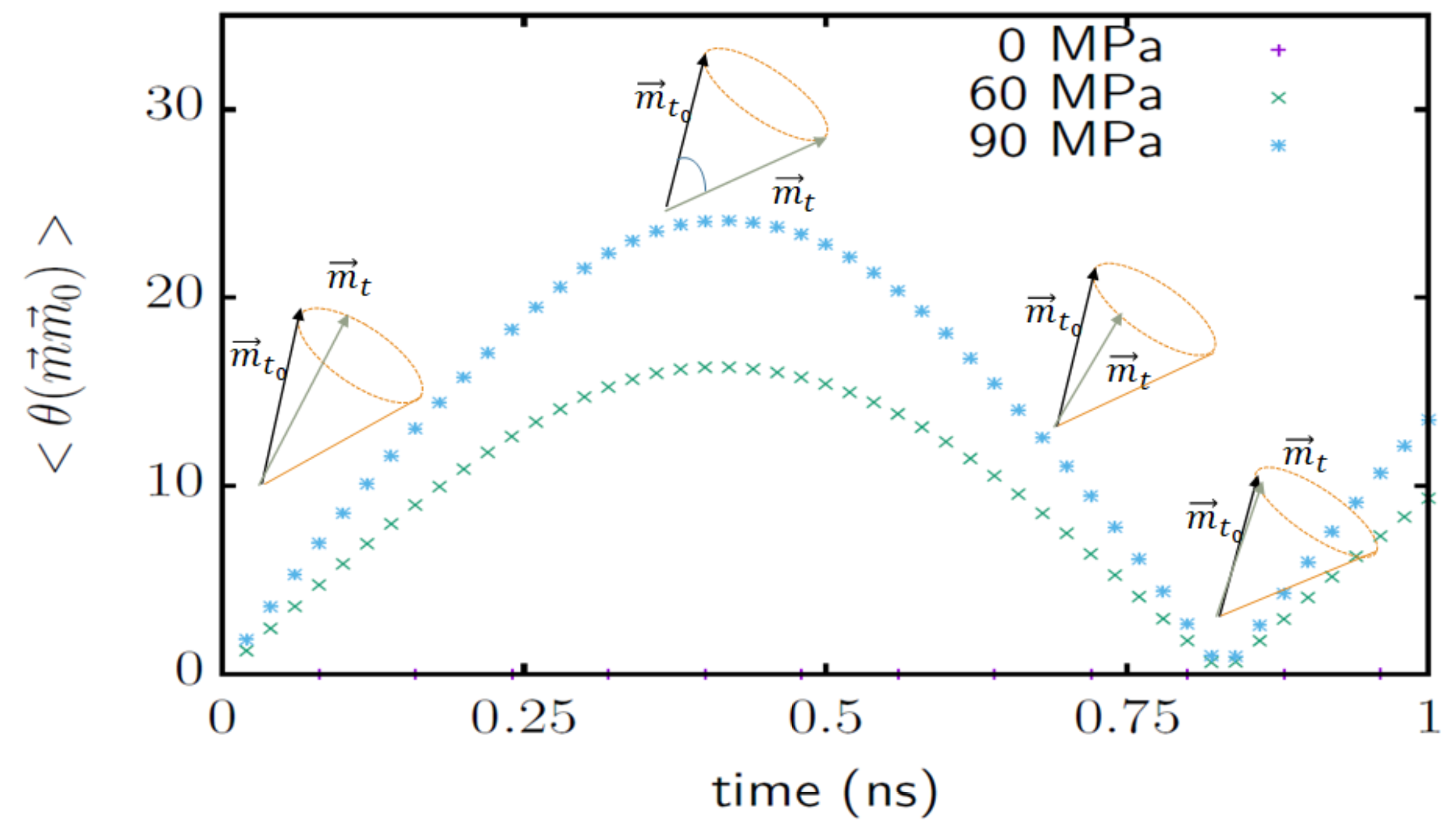


In Fig. S3 we show a schematic representation of how the precession cone is extracted and plotted for different fields in Fig. S4. What we plot in these figures is the averaged angular deviation as a function of time. This represents the spatial average (along the nanostrip) of the angular deviation of the magnetization per numerical cell with respect to the angle of the magnetization in a specific instant. During the numerical evaluation of the AMR loops of the Ni stripe under the action of a SAW, the SAW is acting all the time (thus the magnetization is always evolving). For the estimation of the average precession cone, every time we apply a specific magnetic field, there is first a transitory magnetization motion where the magnetization doesn’t follow a simple precession. After this initial process, the averaged magnetization is stabilized but it is not static, it is precessing due to the action of the SAW. We label t=0 as the instant at which the time averaged magnetization is stabilized (normally within 4 ns after the new magnetic field is applied). This is done for all the figures except for Fig. S4c so this stabilization is visible. Once we have confirmed that the magnetization has stopped evolving to a new configuration and it is only precessing, we start to evaluate the average angle deviation of the magnetization, with respect to the magnetization angle at t=0, as shown in Fig. S3.

**
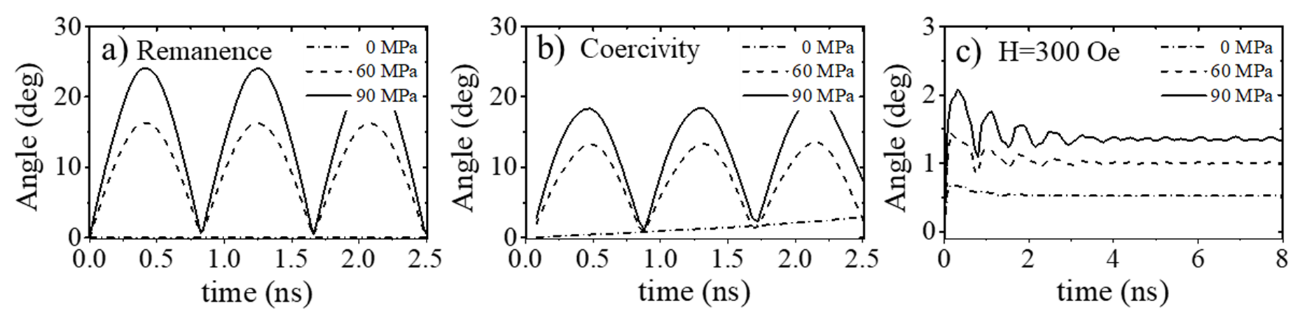
**

**Figure S4**. Time evolution of the spatial average precession angle for (a) remanence, (b) coercive field (c) applied field of 300 Oe, and three different values of the SAW power.

Finally, in the view of the calculation done in the section S1 of this Supplementary Information, in Fig. S5 we show that the components $\sigma_{xz}$ and $\sigma_{zz}$ of the Rayleigh wave, have almost negligible influence in the precession. We ran simulations with only $\sigma_{xx}$ or with only $\sigma_{xz}$ or with only $\sigma_{zz}$. As it can be seen, only $\sigma_{xx}$ is acting on the precession of the magnetization.


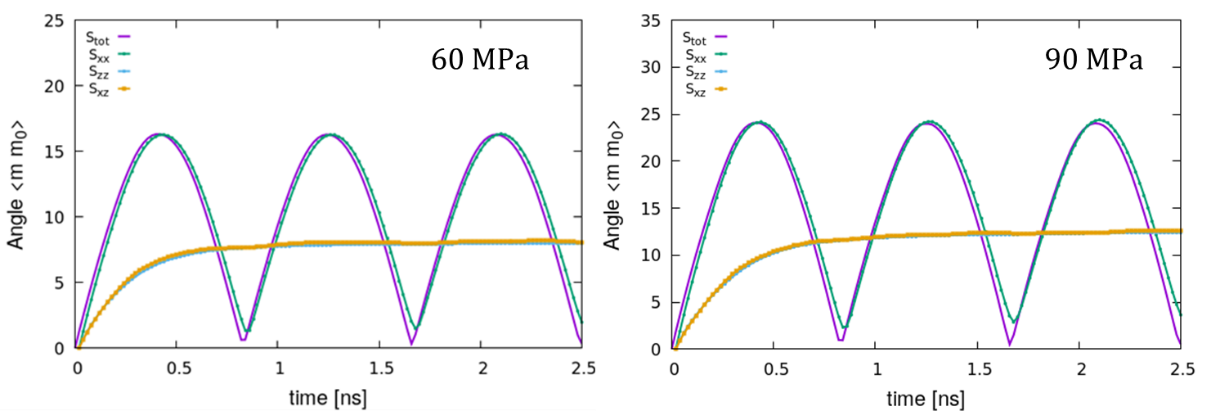


**Figure S5**. Time evolution of the spatial average precession angle for 60 MPa and 90 MPa, using only $\sigma_{xx}$ or only $\sigma_{xz}$ or only $\sigma_{zz}$.

**S4. Spin Pumping Ferromagnetic Resonance on Si/SiO_2_ substrates.**

Here we present the same measurement displayed in Fig. 4 of the main text but using a Si/SiO_2_ non piezoelectric substrate. Obviously, the Ni nanostrip is different to the ones used for the measurements in the main text, as the device had to be fabricated on a different substrate. Also, the experimental rig where this measurement was done, uses a vector network analyser (VNA), rather than a signal generator. It is also performed inside a large electromagnet. This VNA was able to deliver a maximum power of 18 dBm. Beyond the just mentioned small differences from the measurement presented in Fig. 4 of the main text, the important feature is that there is no build up DC voltage in the vertical axis, as seen in Fig. S6. All the plots are symmetric with respect to zero in the vertical axis. This is not the case when the substrate is piezoelectric, as shown in Fig.4 of the main text. This fact and the different shape in the hysteretic section, is a direct evidence that the piezoelectric substrate is contributing to the DC rectified signal.


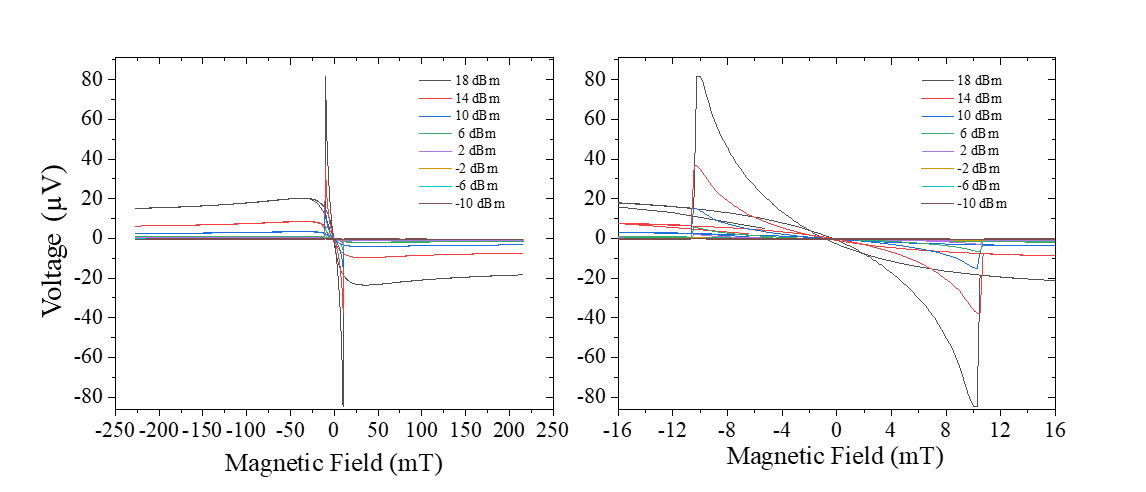


**Figure S6**. Spin Pumping Ferromagnetic Resonance performed on a Ni nanostrip following the set-up displayed in Fig. 4a of the main text, with the external magnetic field pointing along the *y*-axis.

**S5. SP-FMR and Lock-in measurements in the Ni nanostrip with the external magnetic field along the *y*-axis.**

In Fig.S7 we show the Spin Pumping Ferromagnetic Resonance (SP-FMR) measurements for the Ni nanostrip with the external magnetic field applied along the width of the nanostrip (the *y*-axis). Figs. S7b, S7d and S7f are the SP-FMR curves for three selected powers of the SAW. Below each of these curves in Figs. S7c, S7e and S7g, we display the corresponding curve measure with the lock-in set-up, shown in Fig S7h, again with the external magnetic field along the *y*-axis. As it can be seen, the shape of the curves is very similar in both experiments and it is the typical sigmoidal shape for this geometry. The maximum signal is always obtained for positive fields, while in FeCoB (with positive magnetostriction) the maximum is obtained for negative field, indicating the change of 180º phase in the rectified signal.

**
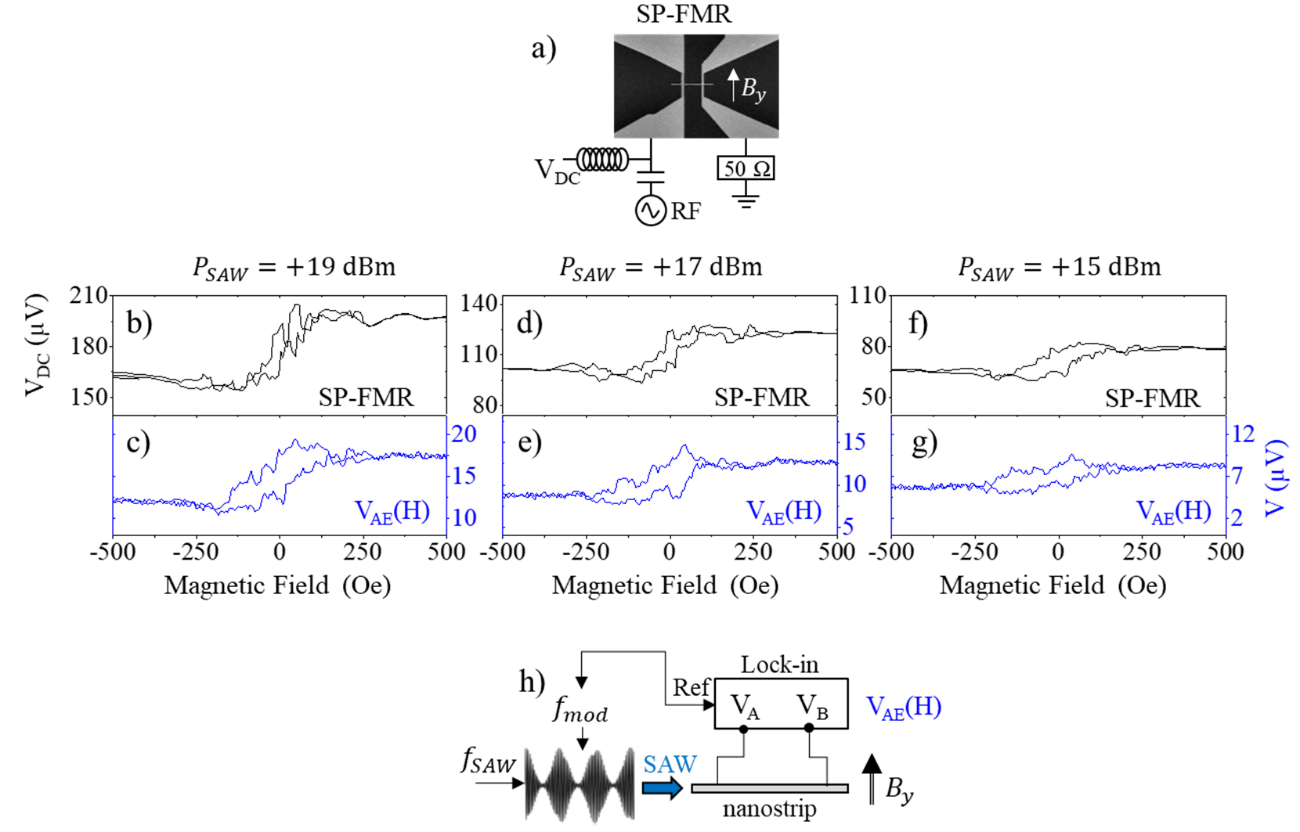
**

**Figure S7**. (a) Show the SP-FMR set up with the external magnetic field along the *y*-axis. (b), (d) and (f) are SP-FMR measurements for different powers of the SAW, as marked on top of the figures. (c), (e) and (g) are the corresponding experiments following the Lock-in set up in (h), where the field is applied also along the *y*-axis.

**References**

1. A. Vansteenkiste, J. Leliaert, M. Dvornik, M. Helsen,F. García-Sánchez, and B. Van Waeyenberge, AIP Advances 4 , 107133 (2014).
2. G. Tang, T. Han, A. Teshigahara, T. Iwaki and K-y Hashimoto. Enhancement of effective electromechanical coupling factor by mass loading in layered SAW device structures. Joint Conference of the IEEE International Frequency Control Symposium & the European Frequency and Time Forum (2015). DOI: 10.1109/FCS.2015.7138870.
3. G. Tang, T. Han, A. Teshigahara, T. Iwaki and K-y Hashimoto. Enhancement of effective electromechanical coupling factor by mass loading in layered SAW device structures. Joint Conference of the IEEE International Frequency Control Symposium & the European Frequency and Time Forum (2015). DOI: 10.1109/FCS.2015.7138870.
4. Piezoelectric coefficients and spontaneous polarization of ScAlN. J. Phys.: Condens. Matter **27**, 245901 (2015).
5. A. Teshigahara, K-y Hashimoto, M. Akiyama. Scandium Aluminium Nitride: Highly piezoelectric thin film for RF SAW devices in multi GHz range. IEEE International Ultrasonics Symposium (2012). DOI: 10.1109/ULTSYM.2012.0481.
